# Reverse Engineering Tone-Deafness: Disrupting Pitch-Matching by Creating Temporary Dysfunctions in the Auditory-Motor Network

**DOI:** 10.3389/fnhum.2018.00009

**Published:** 2018-01-30

**Authors:** Anja Hohmann, Psyche Loui, Charles H. Li, Gottfried Schlaug

**Affiliations:** ^1^Department of Neurology, Heidelberg University Hospital, Heidelberg, Germany; ^2^Department of Psychology, Wesleyan University, Middletown, CT, United States; ^3^Music, Neuroimaging and Stroke Recovery Laboratory, Department of Neurology, Beth Israel Deaconess Medical Center and Harvard Medical School, Boston, MA, United States

**Keywords:** non-invasive brain stimulation, tDCS, pitch matching, auditory-motor network, tone-deafness, singing

## Abstract

Perceiving and producing vocal sounds are important functions of the auditory-motor system and are fundamental to communication. Prior studies have identified a network of brain regions involved in pitch production, specifically pitch matching. Here we reverse engineer the function of the auditory perception-production network by targeting specific cortical regions (e.g., right and left posterior superior temporal (pSTG) and posterior inferior frontal gyri (pIFG)) with cathodal transcranial direct current stimulation (tDCS)—commonly found to decrease excitability in the underlying cortical region—allowing us to causally test the role of particular nodes in this network. Performance on a pitch-matching task was determined before and after 20 min of cathodal stimulation. Acoustic analyses of pitch productions showed impaired accuracy after cathodal stimulation to the left pIFG and the right pSTG in comparison to sham stimulation. Both regions share particular roles in the feedback and feedforward motor control of pitched vocal production with a differential hemispheric dominance.

## Introduction

Making vocal sounds is a fundamental capacity of communication and relies on multiple neural systems that interact to subserve perception, auditory-motor representation, motor plan selection and execution (Hickok and Poeppel, 2007; Pulvermüller, 2010). To acquire and execute vocal-motor plans accurately, the auditory system must represent different dimensions of vocal sound targets (such as loudness, duration and pitch), as well as receive feedback to compute and minimize errors of production considering the intended targets in real time (Guenther, 2006). Many natural languages in the world rely on pitch information to differentiate between specific semantic information. Even in non-tonal languages, the processing of word meaning depends largely on pitch information (Järvikivi et al., 2010). Furthermore, pitch is a reliable cue to aid the resolution of different sound sources, such as different speakers in the same auditory environment (Bregman, 1990). Thus, the ability to perceive pitch information from the environment and from one’s own vocal output, and then to represent this information to accurately produce a target pitch, are important skills that the brain must develop to communicate efficiently using sounds (Fyk, 1985; Stadler, 1990; Kim, 2000).

Neuropsychological, neuroimaging and electrophysiological research in humans and nonhuman primates have identified a network of brain regions involved in pitch perception and production. Electrophysiological recordings in humans have demonstrated that activity in the auditory cortex is suppressed during vocal production (Heinks-Maldonado et al., 2005; Flinker et al., 2010), suggesting that the auditory-motor system builds a precise forward model during sound production. Similar recordings in nonhuman primates have confirmed and extended these findings by identifying neurons with increased sensitivity to one’s own vocal production in the auditory cortex (Eliades and Wang, 2008) and by identifying an auditory region in the posterior insula that responds preferentially to vocal communication (Remedios et al., 2009) as well as a region in the secondary auditory cortex on the anterior-medial STG that seems to be sensitive to pitch information (Norman-Haignere et al., 2013). These results provide support for the role of the auditory core region and parabelt areas in monitoring one’s own vocal production, particularly when pitch information is modulated in the experimental condition.

In humans, functional neuroimaging has shown bilateral superior temporal sulcus (STS), superior temporal gyrus (STG), inferior primary sensorimotor (pre- and postcentral gyrus) and inferior frontal gyrus (IFG) activations during pitch production (i.e., humming a pitched sound vs. a control condition; Ozdemir et al., 2006). A similar pattern of activations was seen by Peck et al. (2009) when subjects were asked to produce a comfortable pitch; additional activations were seen in the putamen, insula and cerebellum during the production of pitches that were higher or lower than the comfortable range (Peck et al., 2009). Zarate and Zatorre (2008); Zarate et al. (2010) showed that the network involved in vocal pitch production depended on experience and expertise, as well as the degree of voluntary control (as manipulated by the task in their study): while perception and production tasks generally activated the posterior superior temporal (pSTG) and STS in the temporal lobe, and the posterior inferior frontal gyri (pIFG) in the frontal lobe, instructions to voluntarily compensate for pitch shifts additionally elicited activity in the cingulate cortex and the pre-supplementary motor area, especially in trained singers (Zarate and Zatorre, 2008; Zarate et al., 2010). Wilson et al. (2010) showed a bilateral frontotemporal network, including the inferior and middle frontal gyri, during singing compared to speech production. Ozdemir et al. (2006) showed that vocal production of “intoned speech” (singing words) showed stronger activation of an auditory-motor network involving the inferior pre- and postcentral gyrus on both hemispheres as well as the superior temporal, and the most inferior portions of the pIFG on the right more than the left hemisphere in comparison to humming (singing a pitch without words; Ozdemir et al., 2006).

In addition to recording brain activity during vocal pitch production in neurotypical individuals, studies investigating individuals who have impaired pitch matching abilities (Loui et al., 2008; Dalla Bella et al., 2009)—i.e., tone-deaf individuals—can also be informative of neural mechanisms underlying pitch production. Structural neuroimaging studies comparing tone-deaf individuals with controls have shown that superior temporal and posterior inferior frontal regions are abnormal in gray matter and in the white matter connections between these regions among tone-deaf people, although the hemisphere most affected in these voxel-based morphometric studies differs between different publications (Hyde et al., 2007; Mandell et al., 2007; Albouy et al., 2013). Furthermore, results from diffusion tensor imaging have demonstrated a marked decrease in connectivity in the arcuate fasciculus (AF), a white matter bundle that connects between superior temporal and inferior frontal areas, among tone-deaf individuals relative to controls (Loui et al., 2009). Loui et al. (2009) found less volume in the AF among 10 tone-deaf individuals compared to 10 non-tone-deaf controls. Using a larger sample size (26 amusics and 26 controls) but different DTI methods Chen et al. (2015) reported some differences in the AF between tone-deaf and control individuals as well as between left and right hemispheres and between different tractography methods, however the between-group differences were not significant at the *p* < 0.05 level. There are many differences between the methodological and theoretical approaches of Loui et al. (2009) and Chen et al. (2015) that might give rise to different results. Loui et al. (2009) used deterministic streamline tractography as implemented in software package MedINRIA; Chen et al. (2015) used probabilistic and deterministic tractography implemented in the software package FSL. These different methods of tractography are known to trade of in sensitivity and specificity (Thomas et al., 2014). Loui et al. (2009) seeded regions of interest between STG and IFG, and between middle temporal gyrus (MTG) and IFG. In contrast, Chen et al. (2015) chose different regions of interest (ROIs) that started with one seed region of interest in the midpoint of the AF (parietal lobe) and conducted probabilistic tractography towards the frontal and temporal endpoints. The location of the frontal lobe ROI is also differed between the two studies: Loui et al. (2009) used the pars opercularis; Chen et al. (2015) used the precentral gyrus, and placed additional exclusion ROIs to identify only the AF. Although their results support their assertion that detection of AF depends on tractography algorithm, Chen et al. (2015) offer no alternative explanation for the auditory-motor deficits in amusia. Thus as Chen et al. (2015) say in their article, “As such, this study [Chen et al.] is not an attempt to precisely replicate prior work given the theoretical (see “Discussion” section above about AF anatomy) and methodological differences”. Since then, Sihvonen et al. (2017) has also shown the importance of the right AF, as well as the inferior fronto-occipital fasciculus (IFOF), in predicting recovery among patients with acquired lesions who score abnormally on tests of aphasia as well as amusia; specifically patients who had intact connectivity in these tracts were better able to recover from acquired amusia.

Taken together, convergent results from animal models, human neuroimaging, and special populations with auditory-motor disorders (e.g., tone-deaf subjects) suggest that the pSTG and pIFG and the connections between them are important nodes in the neural network that enables pitch production and its sensory feedback. However, these reports have relied upon correlational observations of neural activity during the function (or dysfunction) of pitch matching. Using a reverse engineering approach, i.e., selectively disrupting each node in a network to test its resulting function, would provide direct causal tests of each node contribution to a particular function or behavior.

One method to reverse engineer a brain network to examine the causal contributions of a brain region to a particular behavior is to use non-invasive brain stimulation to create a temporary “virtual lesion”. Transcranial direct current stimulation (tDCS) and transcranial magnetic stimulation (TMS) and are two such non-invasive stimulation methods that have been employed to study causal relationships between brain and behavior (Chen et al., 1997; Vines et al., 2006a, b). In auditory studies, TMS has two disadvantages compared to tDCS: first, TMS emits loud clicking sounds during stimulation, which may have confounding effects on the auditory cortex. Second, when applied around the ear, TMS affects the temporalis muscle when applied around the ear, which may contribute to local pain or headaches more than when TMS is applied to other sites (e.g., prefrontal cortex or motor cortex). In contrast, tDCS does not emit any sound. It is not known to affect any peri-aural muscles and is typically well tolerated by the subject, mostly causing only a local tingling or itching sensation in the first few minutes after the stimulation is turned on, which then dissipates during the later phase of a 20–30 min stimulation period. TDCS uses a weak direct current that flows between two cephalic electrodes to modulate levels of regional brain excitability. The direction of current flow between the two electrodes enables the upward and downward regulation of neuronal excitability in targeted cortical regions underlying the electrodes (Nitsche and Paulus, 2000, 2001; Liebetanz et al., 2002; Siebner et al., 2004; Vines et al., 2006a, b, 2008). Cathodal stimulation (i.e., downregulating excitability) can be seen as similar to creating a temporary dysfunction (“virtual lesion”) in the cortical region underlying the electrode location. Blood flow has been shown to be upregulated to a lesser degree in the cathodal conditions compared to the anodal conditions, and blood flow shows differential effects following cathodal and anodal stimulation (Zheng et al., 2011, 2016). Although the stimulation effect is temporary, with behavioral/cognitive effects have been shown to last for about 30 min after a 20–30-min stimulation (Nitsche and Paulus, 2000, 2001; Nitsche et al., 2003; Rogalewski et al., 2004; Ohn et al., 2008), this transient and reversible modulation of cortical excitability thus enables a causal test of the role of the modulated region on a targeted behavior such as pitch matching. To date, studies have shown that cathodal tDCS disrupts reaction time tasks when applied over motor areas, increases auditory frequency-discrimination thresholds after stimulation over Heschl’s gyrus, and causes impairments in pitch memory when applied over the angular gyrus (Vines et al., 2006a, b; Mathys et al., 2010). These results implicate tDCS as a viable method for inducing temporary regional cortical dysfunctions.

While cathodal tDCS when applied to particular regions of the brain or nodes of a network has been shown to cause a dysfunction, anodal tDCS and 35 Hz transcranial Alternating Current Stimulation (tACS) if applied in the same manner has been used to improve short-term memory function for pitched information (Schaal et al., 2013, 2015a,b). To establish the causality of each major node in the neural network, and to test the hemispheric laterality of pitch production functioning, the current study aimed to disrupt the functions of STG and IFG—cortical regions in the hypothesized pitch production network—on either hemisphere and then to observe effects of the modulated functions of each of these candidate regions in a pitch matching task. With tDCS as a neuromodulatory technique, one can tease apart hypothesized roles of several regions in a cortical network by reverse engineering: by systematically modulating each of its nodes, and observing the effects of each modulation on behavior. Our principal aim in the current study is to reverse engineer the auditory-motor network: specifically, to test the causal roles of the pSTG and pIFG in the neural network that subserves pitch matching. As an additional aim, this test allows us to investigate the hemispheric laterality of pitch production. A recent study created a virtual dysfunction in the region around Heschl’s gyrus using cathodal tDCS, suggesting contributions from both hemispheres to pitch discrimination, with effects being more pronounced on the right than the left (Mathys et al., 2010). Further experimental evidence on hemispheric laterality of pitch production function comes from other studies modulating cortical excitability: Wada testing of the non-dominant pSTG resulted in impaired singing in some subjects (Suarez et al., 2010), cooling the right pSTG led to changes in speaking pitch (Katlowitz et al., 2017) and direct intracranial brain stimulation of the right STG also disrupted melody production (Garcea et al., 2017). These studies point towards distinct functions of STG and IFG—cortical regions in the hypothesized pitch production network—that can be modulated by temporary disruptions.

We therefore applied noninvasive brain stimulation over the pSTG and pIFG in each hemisphere to create temporary reversible lesions, and we tested the effects of these localized virtual lesions on pitch matching ability.

## Materials and Methods

### Participants

Fifteen right-handed individuals from the Greater Boston area were recruited via online advertisements and were compensated for participating in this study. Inclusion criteria included: (1) no history of hearing problems or neurological/psychiatric disorders; and (2) a pitch discrimination threshold of less than 5 Hz around the center frequency of 500 Hz. Pitch discrimination was tested using a three-up-one-down adaptive staircase procedure (Cornsweet, 1962; Loui et al., 2008). Mean pitch discrimination threshold was 1.98 Hz (standard error: 0.33 Hz). Subjects had a mean of 7.5 years (range: 0–21 years) of instrumental music experiences, but none of them was a trained singer or a professional musician. All subjects participated in a total of five experimental sessions on five different days. Subjects included seven females and eight males (mean age: 25; range 21–28). The study was approved by the local Institutional Review Board of the Beth Israel Deaconess Medical Center. All subjects gave written informed consent in accordance with the Declaration of Helsinki.

### Procedure

#### Pitch Production Task

At the beginning of the first session for each subject, the subject was asked to hum a tone naturally within his/her vocal range to assess the center of each subject’s comfortable vocal range. Then, subjects were presented with one target tone within their vocal range (ranging from −3 to +3 semitones from the subject’s own produced fundamental frequency) and were asked to reproduce that tone as a practice trial, which was followed by nine experimental trials. Each trial consisted of one sine wave tone within each subject’s vocal range (which ranged from 132 Hz to 262 Hz across subjects), presented through Altec Lansing headphones (AHP512i) at an amplitude of 70 dB and duration of 1000 ms (smooth envelopes with rise and decay times of 50 ms each). The nine target tones had fundamental frequencies of 3, 2, 1, 0.5 and 0 semitones below and above the center of each subject’s comfortable pitch (thus including the subject’s own produced F0). After hearing each tone, subjects’ task was to reproduce its pitch as accurately as possible by humming for 3 s. Vocal production was recorded digitally in Praat (Boersma and Weenink, 2010) via a USB microphone (Logitech 980186-0403 USB Desktop Microphone) for subsequent offline analysis.

#### Transcranial Direct Current Stimulation

**Figure 1:**
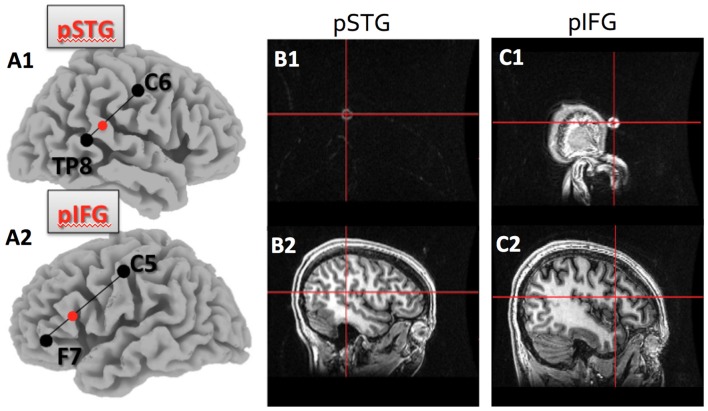
Target location within the posterior superior temporal (pSTG) and posterior inferior frontal gyri (pIFG) as identified by the 10–20 system, and verified using T1 MRI.**(A1)** shows the pSTG target location as 1/3 of the distance between TP8 and C6 on the right side; **(A2)**shows the pIFG target location as 1/3 of the distance between F7 and C5 on the left side; **(B1)** is theMRI marker and crosshair on the skull surface for the pSTG region and **(B2)** is the same crosshair overthe pSTG target; **(C1)** is the MRI marker and crosshair on the skull surface for the pIFG; **(C2)** is the samecrosshair over the pIFG target.

We conducted one session of hummed pitch reproduction before applying 20 min of cathodal tDCS (2 mA), and one session per day on subsequent days to avoid carryover effects between stimulation sessions. During the five different sessions, subjects received cathodal tDCS in each of the four loci (IFG, STG, right and left hemisphere) and the sham condition. The order of stimulation was counterbalanced across subjects. In the session prior to applying non-invasive brain stimulation, all subjects practiced the pitch reproduction task to ensure that they were familiar with the experimental procedures. Based on studies correlating scalp surface markers with high resolution MRI studies, we identified the location of four relevant brain areas in the international 10–20 system, traditionally used for placing electrodes for EEG recordings, whose role in pitch reproduction were of interest (see Figures 1A1–C2 for the locations). The four brain regions were:

1. right posterior superior temporal gyrus (pSTG), which was identified in the international 10-20 system for EEG sites as one third of the distance from TP8 to C6;
2. left pSTG, which was one third of the distance from TP7 to C5;
3. right posterior inferior frontal gyrus (pIFG), which was one third of the distance from F8 to C6;
4. left pIFG, which was one third of the distance from F7 to C5.

In addition to these four brain regions, sham stimulation (described below) was also conducted on one randomly selected region among the four regions identified above.

To verify that the electrode was over the expected region of the cortex, anatomical T1-weighted images of a subset of the subjects were obtained using a 3T GE MRI scanner. The brain regions of interest were identified using the international 10-20 system and a marker was placed on each of the regions. This allowed us to confirm that scalp surface locations identified with help of the 10-20 EEG system identified the pSTG and the pIFG on both hemispheres (see Figure 1).

During the application of cathodal tDCS, the cathodal electrode (using an oval electrode size of 16.3 cm^2^) was placed over the target region, and the reference electrode (a square electrode of 25 cm^2^) was placed over the contralateral supraorbital area, consistent with previously defined stimulation protocols (Vines et al., 2006b). A current strength of 2 mA was applied by ramping up the current from 0 to 2 mA over 30 s, then maintaining the 2 mA stimulation for 20 min, before ramping the current back down to 0 mA (off) over 30 s. For the sham session, the placement of the cathodal electrode was counterbalanced between subjects among one of the four target regions, while the reference electrode was again over the contralateral supraorbital area. Sham stimulation was done by ramping up the current from 0 to 2 mA over 30 s, then ramping the current back down to 0 mA (off) for the next 30 s, and then leaving the stimulation off for the remaining 20-min period. This procedure has been previously used in other studies (Gandiga et al., 2006). All participants reported a tingly sensation or a slight, heated prickly sensation under the cathodal and/or reference electrode with ramping up of the current at the beginning of the stimulation. This sensation was the same for cathodal as well as for sham stimulation and faded away after approximately 1 min. Participants were unable to distinguish whether they received sham stimulation or real stimulation according to post-experiment interviews. Order of stimulation was counterbalanced across subjects.

### Data Analysis

Subjects’ vocal production was recorded and pitch-extraction was applied offline using the autocorrelation method in Praat (Boersma and Weenink, 2010). Since there were fluctuations in fundamental frequency within each trial, and stimulation was predicted to have the greatest effect on initial vocal-motor planning and preparation, only the F0 values of the first 500 ms of each production were averaged and analyzed for each of the nine produced pitches per subject. For statistical analysis, all frequencies were converted from absolute frequency (in Hertz) to relative deviation from target frequency in cents of a semitone (100 cents = one semitone) using the following formula:

(1) $$\text{CD = 1200 * }{\text{log}}_{\text{2}}({\text{F0}}_{\text{produced}})\;-\;{\text{log}}_{\text{2}}({\text{F0}}_{\text{target}}),$$

where CD is Cents Deviation, F0_target_ is the target fundamental frequency and F0_produced_ is the produced fundamental frequency. Change scores in cents deviation for stimulation relative to sham were computed as:

(2) $$\%\text{change = }({\text{CD}}_{\text{stimulation}}\;-\;{\text{CD}}_{\text{sham}}){\text{/CD}}_{\text{sham}}\text{ * 100}\%$$

Cents deviation and percentage change scores were exported to SPSS for statistical analysis.

## Results

**Figure 2:**
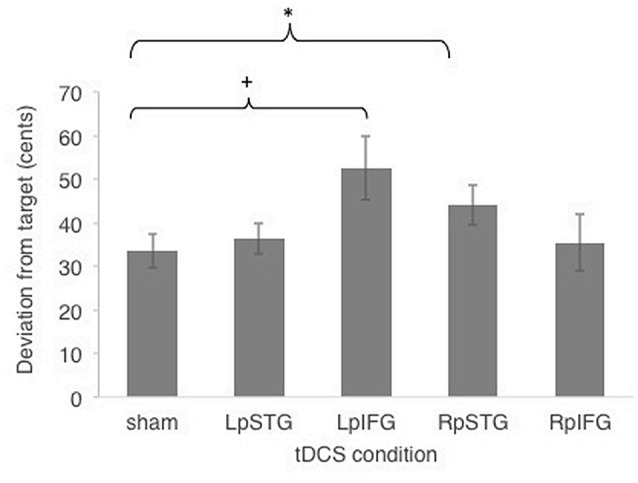
Effects of cathodal and sham transcranial direct current stimulation (tDCS) stimulation on cents deviation from target. Error bars reflect between-subject standard error. *p < 0.05 (Bonferroni-corrected); ^+^*p* < 0.05 (uncorrected).

Subjects were generally accurate at the task, with mean cents deviation from target frequency being less than one semitone across all stimulation conditions (*M* = 40.4 cents, SE = 5.15 cents). Figure 2 shows effects of different stimulated regions on cents deviation from target frequency. Cents deviation was lowest for the sham condition (Mean = 33.47, SD = 14.83) and highest in the LpIFG condition (Mean = 52.65, SD = 28.59), followed by RpSTG (Mean = 44.075, SD = 17.64), LpSTG (Mean = 36.38, SD = 13.54) and RpIFG (Mean = 35.44; SD = 25.07).

A repeated-measures analyses of variance (ANOVA) on the dependent variable of cents deviation with the independent variables of stimulation site (Five levels: LpSTG, LpIFG, RpSTG, RpIFG, sham) showed a significant effect of tDCS site (*F*_(4,56)_ = 2.719, *p* = 0.039, partial *η*^2^ = 0.16).

Follow-up pairwise comparisons of real stimulation compared to sham stimulation revealed a significant difference between the right pSTG stimulation (*t*_(14)_ = 2.21, *p* = 0.044) and sham stimulation, as well as between the left pIFG compared to sham (*t*_(14)_ = 2.85, *p* = 0.012). The latter survives Bonferroni correction across the four tested regions. Because the variability was not normally distributed, we also ran non-parametric tests on pairwise comparisons between sham and real stimulation for each region. Non-parametric tests showed the same significantly higher deviation for LpIFG stimulation compared to sham (Wilcoxon Signed Ranks Test, *Z* = 2.726, *p* = 0.006, surviving Bonferroni correction across four regions) and a significantly higher deviation for RpSTG compared to sham (*Z* = 1.99, *p* = 0.047, not surviving *post hoc* correction).

**Figure 3:**
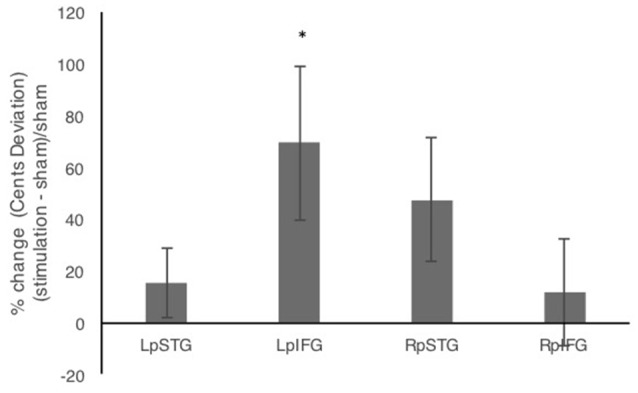
Percent change after stimulation relative to sham condition. Error bars reflect between-subject standard error. *p < 0.05.

In addition to comparing mean deviations from target frequency in cents, we tested % change relative to sham (Figure 3). Repeated measures analysis of variance (ANOVA) comparing % change in cents deviation during the four stimulation conditions relative to sham showed a significant effect of stimulation site (*F*_(3,42)_ = 3.192, *p* = 0.033, partial *η*^2^ = 0.186). One-sample *t-tests* against the chance level of 0% showed significant effects of stimulation relative to sham for LpIFG (*t*_(12)_ = 2.35, *p* = 0.037) and a marginally significant effect for RpSTG (*t*_(12)_ = 1.99, *p* = 0.07).

The size of the pitch discrimination threshold, a baseline control for pitch discrimination ability assessed before the first stimulation session, was not correlated with the average effect of stimulation (*r* = 0.32, n.s.). Average cents deviation from target frequency showed no significant correlation with number of years of musical training (*r* = 0.12, n.s.), suggesting that the minimal to moderate amount of instrumental musical training in our group o subjects (without any professional music background) did not affect pitch matching performance.

## Discussion

The present study used cathodal-tDCS to test the causal role of several cortical regions in pitch matching. Results showed that temporarily disrupting the left pIFG and to a lesser degree the right pSTG decreased accuracy in pitch matching performance.

The posterior IFG and posterior STG have previously been shown to play an important role in pitch production and vocal pitch regulation (Zarate and Zatorre, 2008; Peck et al., 2009; Wilson et al., 2010). In addition, these regions are structurally abnormal in gray matter concentration and cortical thickness in both right and left hemisphere regions among individuals who have problems matching a pitch or singing in tune with others, a disorder commonly referred to as tone-deafness (Hyde et al., 2006, 2007; Mandell et al., 2007). Tone-deaf individuals also have poor singing ability, specifically higher deviation during pitch matching tasks (Loui et al., 2008; Dalla Bella et al., 2011; Williamson et al., 2012; Yang et al., 2014; Loui, 2015) and both of these regions are critically important in the control of pitch while singing. Functional importance of these regions is further supported by studies on acquired amusia after stroke, that show persisting pitch-processing deficits after lesions to the right STG and IFG (Sihvonen et al., 2016), and the recovery of musical functions over time after lesions to the left IFG (Sihvonen et al., 2017). The present approach links together these lines of evidence by disrupting intrinsic cortical activity of several regions in a network, and testing for the effects in a pitch matching behavior. Our results show the most significant reduction in pitch matching ability after left pIFG stimulation, suggesting a causal role of left pIFG in pitch matching, and further demonstrating that by reverse engineering the auditory-motor network, we can effectively simulate an aspect of tone-deaf-like behavior.

Pitch production ability was measured by mean deviation, in cents of a semitone, of the produced fundamental frequency from the target fundamental frequency. This objective measure, derived from acoustic analyses of recorded pitch productions, is a reliable index of how far subjects’ vocal production deviated from a given target pitch (Loui et al., 2008; Dalla Bella and Berkowska, 2009; Dalla Bella et al., 2009). The present pitch production paradigm provides a sensitive measure of pitch matching, an important aspect of singing ability, in a controlled environment, and is consistent with existing protocols for singing assessment (Demorest et al., 2015). Subjects are generally accurate at the task, generally producing within one semitone of the target fundamental frequency. Notably, even after tDCS subjects are able to reproduce the general direction of pitch height and do not reach a tone-deaf level of performance. Although the effects of tDCS are consistent across subjects, they are still subtle and the effects we have induced may not approach the level of impairment in tone-deaf subjects.

The effects of cathodal stimulation compared to sham stimulation were most pronounced for stimulation over the left pIFG, followed by right pSTG, with no significant effects observed after stimulation over the left pSTG or the right pIFG. Although previous findings have generally shown a preponderance of the right hemisphere for pitch-related functions (Zatorre et al., 2002; Mathys et al., 2010), the fact that both hemispheres were affected by the stimulation is not surprising given the current pitch production task, as a bi-hemispheric role in the execution and sensorimotor control of vocal production for both speaking and singing has been supported by various studies (Brown et al., 2004; Ozdemir et al., 2006). Another possible explanation why the effects were less pronounced on the right side might be that the non-dominant hemisphere is in general more robust against interference with stimulation. Support for this hemispheric difference comes from TMS over the left and right IFG, in which disruption in speaking was observed after stimulation over left IFG stimulation, but not over the homologous right IFG (Stewart et al., 2001).

Reducing excitability in left pIFG and right pSTG independently impaired subjects’ pitch-matching performance. This might be due to two different underlying functions of those regions: theoretical models of sound production suggest that while the left pIFG is more involved in sound-motor planning, the pSTG is more involved in perceiving the target pitch and generating a mental representation of the sound to be produced (Tourville and Guenther, 2011). Results converge with investigations of disordered singing in behavioral (Loui et al., 2008; Dalla Bella and Berkowska, 2009; Dalla Bella et al., 2009) as well as neuroimaging studies (Hyde et al., 2007; Mandell et al., 2007; Loui et al., 2009), and evidence from studies creating temporary disruptions in singing performance (Suarez et al., 2010; Garcea et al., 2017; Katlowitz et al., 2017), all of which support a multi-regional network of brain areas, centering around pSTG and pIFG, in pitch perception and production. The present results further narrow down the causal roles of these different regions into distinct stages of the pitch matching task establishing a prominent role for the LpIFG and to a lesser degree for the RpSTG in sound-motor mapping. The pattern of our results suggests that RpSTG helps in perceptually representing the sound target, such that its disruption results in a badly represented sound target, which in turn leads to higher deviation. In contrast, LpIFG helps in sound-motor mapping, such that its disruption causes more variable production as well as an inability to match the target. Both pSTG and pIFG were causally involved in targeting and fine-tuning pitch production.

## Concluding Remarks

Taken together, results suggest that non-invasive brain stimulation can be used to reverse engineer a disorder with a suspected cortical dysfunction. By disrupting performance in a pitch production task after inducing virtual lesions via noninvasive brain stimulation, the present experiment provided causal evidence for the role of the left pIFG and right pSTG in pitch production. Results shed light on the different stages of the auditory-motor neural network that maintains control of speech production and communication, and have clear implications for targeting future rehabilitative strategies for improving the prosodic content of speech production in populations with communication disorders.

## Author Contributions

AH, PL, CHL and GS: substantial contributions to the conception or design of the work; or the acquisition, analysis, or interpretation of data for the work; drafting the work or revising it critically for important intellectual content; final approval of the version to be published; agreement to be accountable for all aspects of the work in ensuring that questions related to the accuracy or integrity of any part of the work are appropriately investigated and resolved.

## Conflict of Interest Statement

The authors declare that the research was conducted in the absence of any commercial or financial relationships that could be construed as a potential conflict of interest.
